# Supplementary material for: The interactome of intact mitochondria by cross-linking mass spectrometry provides evidence for coexisting respiratory supercomplexes
Source: Mol Cell Proteomics. 2017 Dec 8;17(2):216–32. doi: 10.1074/mcp.RA117.000470 (PMC5795388; doi:10.1074/mcp.RA117.000470)
Supplement: Supplemental Data [file supp_RA117.000470_133922_0_supp_23978_fzffwf.zip › spectra_annotation/mito_spectra_annotation/170-1-8-1-12-1.pdf]

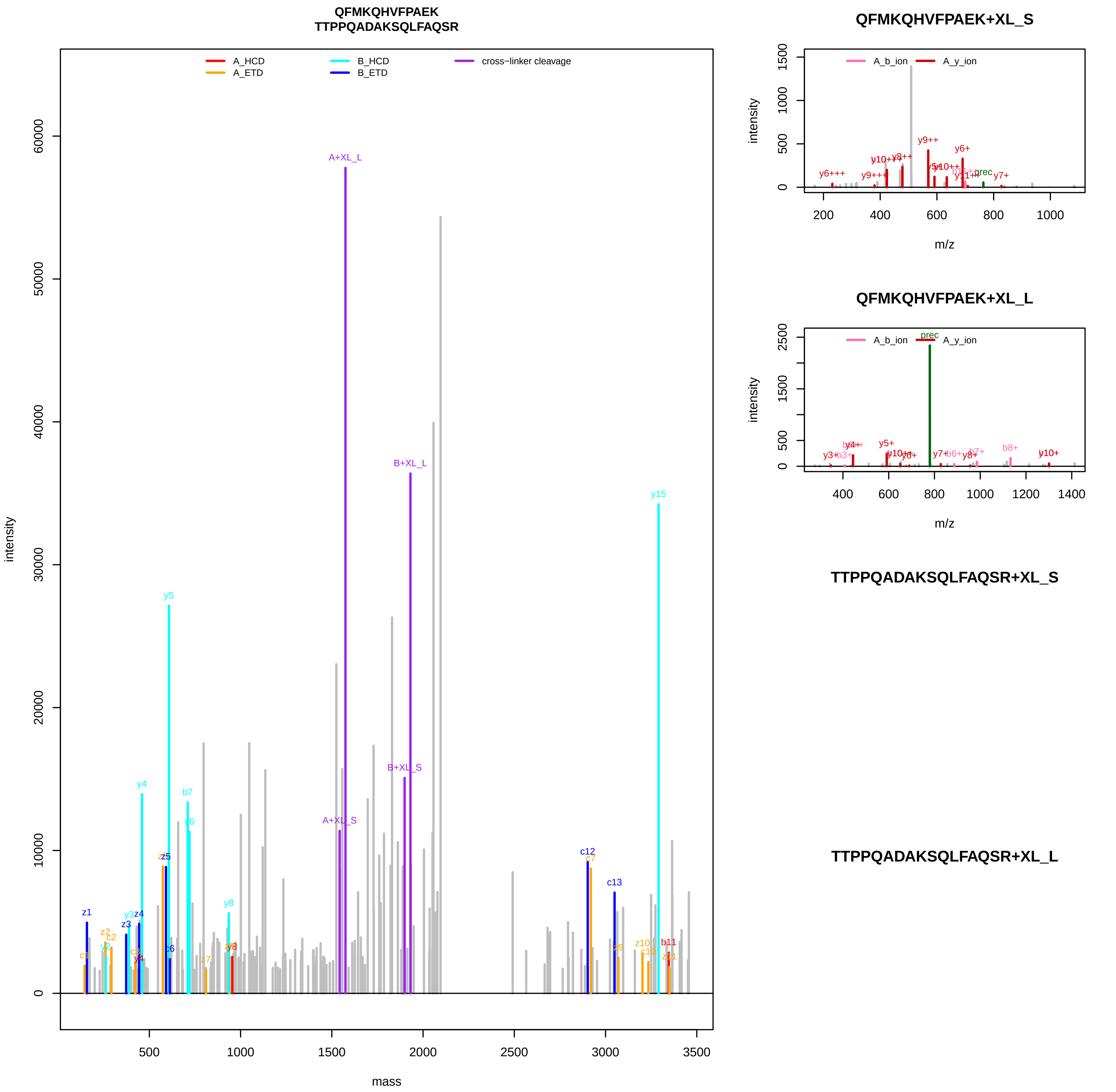
QFMKQHVFPAEK
TTPPQADAKSQLFAQSR
A_HCD
A_ETD
B_HCD
B_ETD
cross−linker cleavage
intensity
mass
A+XL_L
B+XL_L
B+XL_S
A+XL_S
y15
y5
y4
b7
y6
y8
c12
c7
c13
z1
z3
z4
z5
c6
QFMKQHVFPAEK+XL_S
QFMKQHVFPAEK+XL_L
TTPPQADAKSQLFAQSR+XL_S
TTPPQADAKSQLFAQSR+XL_L
A_b_ion
A_y_ion
m/z
prec
y9++
y6+
y6+++
y9+++
y10++
y8++
y7+
y5+
y4+
y3+
y10+
b8+
